# Implementation of the WHO Standardized Emergency Unit Form: Trauma at Bashair Teaching Hospital

**DOI:** 10.7759/cureus.75804

**Published:** 2024-12-16

**Authors:** Khalid Salih, Ahmed Abdelrahman, Ali E Mohamed, Idriss Tahir

**Affiliations:** 1 Human Clinical Anatomy, National University, Khartoum, SDN; 2 Medicine, University of Khartoum, Khartoum, SDN; 3 General Surgery, Bashair Teaching Hospital, Khartoum, SDN

**Keywords:** audit, documentation, implementation, quality improvement project, trauma, who emergency unit form

## Abstract

Aim​​​​

The aim is to audit the documentation process for trauma patients presenting to the surgical trauma department and to implement the WHO Standardized Emergency Unit Form: Trauma at Bashair Teaching Hospital in Khartoum, Sudan, in 2022.

Methodology

The audit was commenced by reviewing the documentation method for trauma patients. There was no standardized form in use. Current practice feedback was collected; then, the WHO Standardized Emergency Unit Form: Trauma was tested. The form was then implemented in one unit, where sessions for training on the proper use were conducted. Successively, generalization in all surgical units was achieved. From the hospital's records, comparisons were made between complications, mortality rates, duration of hospital stay, and mean time to initial assessment before and after execution.

Results

Case coverage was the primary indicator, describing the percentage of cases documented using the form per week. In the first cycle, the first week scored 31%, improving to 67% following that. The second cycle had 84-91% coverage throughout. The last cycle faced doctor strikes in the first week and the start of a new shift of foundation year trainees, achieving 53%, which was restored to 78% in the succeeding week. Furthermore, the mean time to initial assessment was significantly reduced by 31%, decreasing from 39 minutes±7.2 to 27 minutes±5.8 (p=0.023).

Conclusion

Medical documentation is crucial for patient care, ensuring continuity and addressing medico-legal issues. Implementation of the form improves communication between healthcare providers and ensures a systematic method to approach trauma patients, thereby warranting high-quality care.

## Introduction

Good medical documentation forms the backbone of medical care, specifically in facilitating clear communication between healthcare professionals, which is vital for providing continuity of care [1].

In trauma care, a pertinent type of medical documentation is the trauma form. It is important that this document be exhaustive to ensure support across different settings and professionals. Additionally, trauma forms have significant medico-legal implications as they provide vital evidence in various contexts [2,3].

Several techniques have been developed to ensure optimal healthcare delivery for trauma patients, including a standardized trauma form. This allows a structured approach to the primary survey, prevents missed assessments, and serves as an analysis tool for quality improvement (QI) [4].

As is the case with most hospitals in low-resource settings, Bashair Teaching Hospital lacked a standardized trauma registry. This project aimed to audit trauma documentation and improve consistency in the hospital’s emergency surgical department.

In line with established clinical protocols, the "WHO Standardized Unit Form: Trauma" was introduced for use by the surgical unit staff. This form includes socio-demographic information, primary/secondary surveys from advanced trauma life support, chief complaints, vital signs, medical history, physical exams, diagnostic tests, interventions, management plans, and reassessments [5].

Accurate documentation in trauma care is necessary for various reasons: (a) to facilitate communication among healthcare providers; (b) to provide a record of the patient’s treatment, reducing errors; and (c) to address medico-legal concerns. Detailed documentation enhances decision-making and patient outcome monitoring. Standardized forms, such as the WHO Standardized Emergency Unit Form: Trauma, ensure a systematic approach to recording trauma history and management [6].

Technological advancements, such as Trauma Tracker, have improved documentation quality. Montagna et al. (2019) reported significant improvements in documentation, reduced mortality, and enhanced trauma team efficiency with automated data collection, underscoring the importance of record-keeping for better outcomes in trauma care [7].

In low-resource settings, standardized documentation, such as the WHO Standardized Emergency Unit Form: Trauma, is particularly beneficial. The WHO supports this approach as part of QI programs to address gaps in trauma care globally. These programs have been successful in high-income countries and are being adapted for low- and middle-income countries (LMICs) [6].

Standardized forms allow trauma teams to follow a structured process for primary and secondary surveys, ensuring accurate data collection and communication. The WHO has shown that formal documentation in QI programs improves patient outcomes by promoting standardized recording during trauma resuscitation and handovers [6].

Triage and documentation are crucial for trauma care management. Granström et al. (2018) stated that systematic triage protocols help ensure optimal trauma care based on physiological status, injury characteristics, and trauma mechanisms. Using criteria-based protocols like the WHO Standardized Emergency Unit Form: Trauma improves documentation accuracy, reduces over- and under-triage, and optimizes resource use [8].

In addition to preserving care continuity and quality, accurate medical documentation serves as a safeguard against medico-legal risks. Structured forms like the WHO Standardized Emergency Unit Form: Trauma enhance the accuracy of clinical records and ensure timely interventions. Kottenstette et al. (2019) reported that trauma-informed assessments (TIA) improved documentation and referral rates, highlighting the importance of understanding trauma history for better outcomes [9].

In settings where trauma care is fragmented and data collection is challenging, standardized documentation systems like the WHO Standardized Emergency Unit Form: Trauma are essential. Bommakanti et al. (2018) highlighted that hospitals in LMICs face challenges related to data quality, resources, and inadequate prehospital care. Despite these challenges, trauma registries are increasingly recognized as vital tools for QI in LMICs, reflecting their success in high-income countries [10].

## Materials and methods

**Figure 1:**
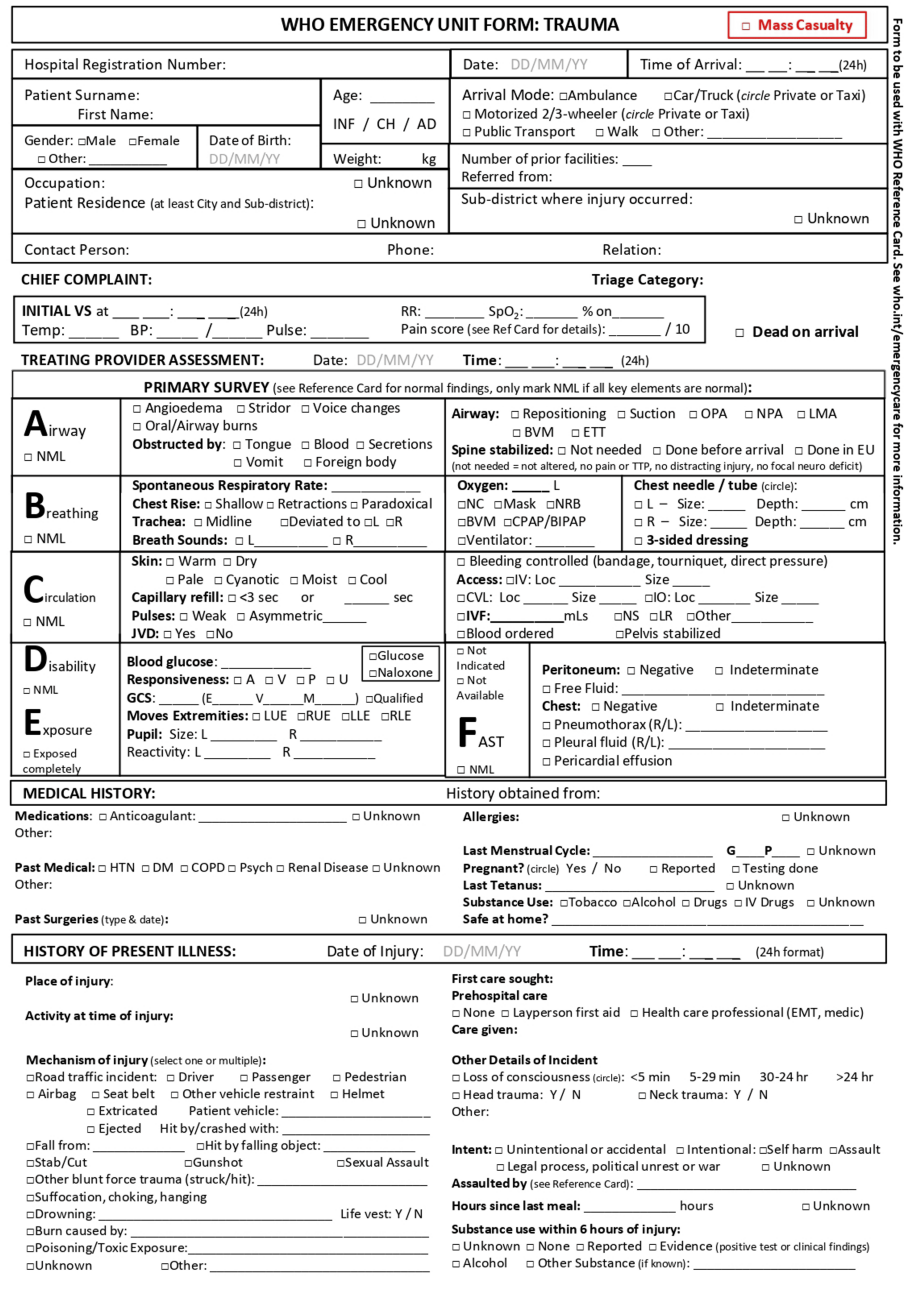
WHO Standardized Emergency Unit Form: Trauma - Page 1

**Figure 2:**
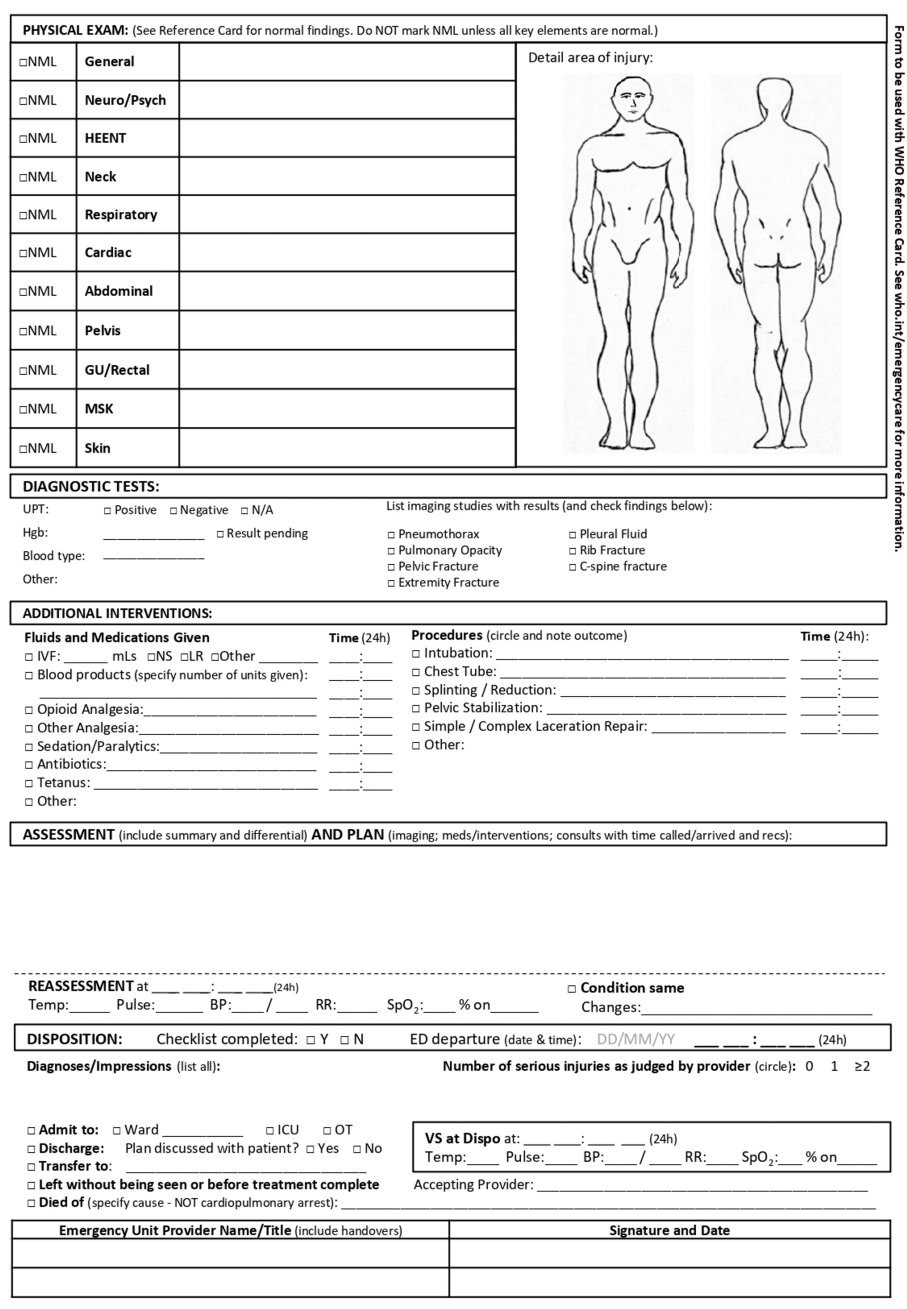
WHO Standardized Emergency Unit Form: Trauma - Page 2

Initially, the documentation method for trauma patients in the emergency room was evaluated at Bashair Teaching Hospital. There was no standardized form mandated by the hospital’s guidelines for practice, and the registry solely contained the sociodemographic information of the patients. Part of the staff was using prescription sheets to document vital signs, important points of the patient’s history, and the suggested management plan. On the other hand, some practitioners were dependent on their memory and recall in dealing with their patients. Additionally, the mortality rate, average length of hospital stay, and mean time to initial assessment for the past month were noted. This data was collected from the hospital’s records in the Department of Medical Records and Statistics. Moreover, doctors’ feedback on the current practice, including assessment, intervention, and documentation, was gathered. The majority of respondents conveyed discontent and stated that they struggled with the unavailability of a proper tool for documentation and expressed pleasure with the idea of possible change. “WHO Standardized Emergency Unit Form: Trauma” (Figures 1, 2) [5] was then trialed for applicability. 

First cycle

In this stage, one unit of the surgical department was included. First, a meeting of all the unit staff was carried out, in which education and training sessions for proper use of the form were accomplished. The sessions were supervised by the specialist, and the unit members were subdivided into groups. The “Reference Card for WHO Emergency Unit Form: Trauma” was used as a guide, and practical applications were performed by each member [11]. Then, the form was implemented for two weeks. At the end of each week, forms used were collected and analyzed as follows: the percentage of the documented cases was calculated from the total number of presenting patients, then each segment was assessed, and the corresponding percentage of completion from the case coverage was deducted. Moreover, reflection slips were distributed, and feedback was gathered, aiming to tackle any difficulties faced while using the form. 

Second and third cycles

The form was generalized in all surgical units. As stated previously, education and training sessions were carried out, with an emphasis on how to approach obstacles encountered by the pilot unit. Sequentially, the form was implemented throughout the department, after which the data was collected and analyzed.

The mortality rate, average length of hospital stay, and mean time to initial assessment were reported for the month following the conclusion of the project and the establishment of the form as the department’s official documentation method. This data was compared with that collected prior to launch to evaluate the change.

## Results

The first variable to be measured was case coverage, which is the main indicator of success for this project. In simple terms, it describes the percentage of cases that were documented using the implemented form from the total number of patients presenting per shift, keeping in mind the aim for total coverage. In the first cycle, the first week’s result was the lowest of all, 31%. This was partly due to the hesitancy of staff to participate and perform with motivation. Improvement was noted in the second week, with 67%. The second cycle was the best, almost reaching the goal, scoring 84-91% coverage in subsequent weeks. The last cycle faced many limitations, including a strike of some units due to improper conditions and the subsequent closure of the surgery department on some occasions, as well as the rotation of house officers (foundation trainee equivalent). This led to a substantial drop in the first week, scoring 53% overall, with improvement in the following week to 78%.

The mortality rate, which was 3% beforehand, displayed a decline to 2% following the project. The average length of hospital stay has shifted from 6.5 days to 5.8 days, and the mean time to initial assessment was altered from 39 minutes±7.2 to 27 minutes±5.8 (p=0.023). Moreover, doctors’ feedback on the change from previous practice was satisfactory.

The results of the remaining variables are described in the following table, expressed as percentages acquired from the total of cases covered by the form (Table 1).

**Table 1:** Percentage of Documentation/Weeks 1-6

Variable/week	1	2	3	4	5	6
Case coverage	31	67	84	91	53	78
Sociodemographic data	82	85	76	94	78	92
Vitals	79	88	93	98	94	100
Chief complaint	75	83	90	86	84	92
Primary survey	71	84	79	88	82	97
Medical history	65	72	69	65	75	80
History of presenting illness	73	86	77	89	88	93
Examination	81	87	83	90	85	96
Diagnostic tests	78	89	73	88	87	92
Interventions	69	84	76	90	81	87
Assessment and plan	72	90	79	83	85	94
Re-assessment	57	75	66	72	83	87

## Discussion

This QI project supports the use of standardized documentation tools like the WHO Standardized Emergency Unit Form: Trauma for improving trauma care even in resource-poor environments [12]. The form is accompanied by a reference card, which helps clinicians use the form appropriately and ensures data quality. The WHO Standardized Clinical Form not only improves care quality but also provides a simple method for capturing important data points [11]. According to NICE guidelines, structured information recording in major trauma is crucial. This includes noting fatal bleeding, airway management with spinal immobilization, breathing, circulation, neurological disability, and full exposure as part of the primary survey. Additionally, one trauma team member should be responsible for real-time documentation, with the trauma leader verifying completeness [13].

The improvement in case coverage, despite initial challenges, highlights the utility of a multifaceted implementation approach. Staff training, role clarity, and visual aids improved form use. The 31% decrease in time to assessment demonstrates a direct impact on patient care, with potential implications for morbidity and mortality [14]. However, attributing these improvements solely to the form is premature; further research is needed to control for confounding variables.

Long-term sustainability faces several constraints, such as staff turnover, as seen in the third audit cycle. Continuous training, especially for new staff, is essential to maintaining improvements. Expanding the audit to assess documentation accuracy, completeness, and its impact on communication between healthcare providers is necessary. Gathering feedback from both patients and staff on the form’s usability would provide a broader understanding of its impact. Studies show that inadequate documentation affects audits, research, and legal issues, while trauma chart templates enhance documentation quality [15].

This project aligns with findings from Montagna et al. (2019), who showed that electronic trauma documentation systems reduce bias and errors, highlighting the importance of technology in fast-paced environments where manual documentation may lead to mistakes [7]. The Trauma Tracker system demonstrated the benefits of real-time tracking, automated reports, and improved data capture. These features reduce the trauma team’s documentation burden and aid decision-making during trauma management, leading to improved outcomes.

This initiative also supports the WHO’s global recommendations to improve trauma care through better documentation and quality monitoring. The WHO standardized trauma form is essential for improving trauma care organization and service delivery. Systems such as morbidity and mortality (M&M) reviews form a key component of trauma QI programs [6]. Addressing gaps in the WHO guidelines through staff training and structured documentation has been shown to reduce preventable trauma-related deaths, even in low-resource settings. The reduction in time to assessment aligns with the WHO’s findings on how structured documentation improves workflows and outcomes [6].

Challenges like staff turnover and external disruptions (e.g., doctor strikes) were noted, which the WHO recognizes as barriers to sustaining QI projects. Continuous education and retraining are vital for sustaining the benefits of implementing the form [6]. Granström et al. (2018) emphasized the importance of structured triage and documentation protocols for patient safety, particularly in reducing over-triage and under-triage rates. The success of these protocols in Sweden supports the use of tools like the WHO trauma form, which in our audit led to reduced time to assessment and improved form utilization [8]. However, ongoing evaluation of triage accuracy and staff compliance is needed to maintain these benefits, as challenges like staff turnover and documentation inconsistencies could undermine progress [8].

This QI project also aligns with trauma-informed care (TIC) models, which emphasize structured documentation for improving service delivery and identifying patient needs. Kottenstette et al. (2019) found that TIA improved documentation quality and referral rates. Integrating TIC principles with trauma documentation, as demonstrated in our audit, suggests the potential for further improvements in care efficiency and quality [9].

The challenges encountered during WHO trauma form implementation in this audit mirror those described by Bommakanti et al. (2018) in trauma registry implementation in LMICs. Barriers like limited resources, staff turnover, and data quality issues were addressed through stakeholder engagement, adapting systems to local contexts, and continuous training. These strategies were crucial in overcoming obstacles and improving documentation accuracy and completeness [10]. Consistent with trauma registries, our audit showed better documentation led to improved case coverage and reduced time to assessment [10].

Previous studies showed a significant reduction in hospital stay length and a 10-fold increase in hospital profit after implementing standardized trauma documentation [16]. In a low-income country hospital, introducing a trauma registry and electronic patient registration system led to a 20.9-fold increase in completed trauma documentation [17]. Pennsylvania trauma centers reported success in improving emergency department documentation through education, redesigned forms, and peer review. Post-implementation, fewer documentation deficiencies were noted, with benefits in identifying low-severity injuries and administrative involvement [18]. These strategies, alongside staff engagement, are key to sustaining trauma documentation improvements.

## Conclusions

The implementation of the form was vulnerable to several factors that affected overall patient coverage. The most significant of these was the rush of patients in the ER, which created an obstacle to time management and hindered the achievement of full coverage. With further practice and time, the response improved significantly, leading to a substantial increase in the documentation of cases. This, in turn, contributed to better assessments by doctors and, subsequently, an improvement in the quality of care provided by the department. This outcome reflects the vitality of such QIs, which, if organized and incorporated into the healthcare system, will lead to measurable differences in patient care.
